# Enhancing your delivery of cognitive therapy for PTSD: a celebration of the work of Hannah Murray

**DOI:** 10.1017/S1754470X24000370

**Published:** 2024-11-29

**Authors:** Graham R. Thew, Emma Warnock-Parkes, Sharif El-Leithy, Kerry Young, Nick Grey, Kirsten V. Smith, Gabriella Tyson, Firoozeh Daraei, Jennifer Wild, Belinda Graham, Richard Thwaites, Sophie Grant, Alice Kerr, David M. Clark, Anke Ehlers

**Affiliations:** 1 Department of Experimental Psychology, University of Oxford, Oxford, UK; 2 Oxford Health NHS Foundation Trust, Oxford, UK; 3 Institute of Psychiatry, Psychology and Neuroscience, King’s College London, London, UK; 4 Traumatic Stress Service, South West London and St George’s Mental Health NHS Trust, London, UK; 5 Woodfield Trauma Service, Central and North West London NHS Foundation Trust, London, UK; 6 Sussex Partnership NHS Foundation Trust, UK; 7 Newham Talking Therapies, East London NHS Foundation Trust, UK; 8 Cumbria, Northumberland, Tyne and Wear NHS Foundation Trust, UK

## Introduction

In December 2023 we sadly lost our friend and colleague Dr Hannah Murray, who passed away peacefully after a long illness. Hannah was a dedicated clinical psychologist and researcher, passionate about improving the treatment of post-traumatic stress disorder (PTSD) and supporting clinicians to develop their skills in trauma-focused cognitive therapy (CT). Hannah’s work spanned multiple topics within PTSD and cognitive therapy. She leaves an impressive legacy of clinical guidance papers, books and videos that will continue to support current and future practitioners to develop their practice.

Hannah was a frequent contributor to the British Association for Behavioural and Cognitive Psychotherapies (BABCP), publishing articles in its journals and delivering training both in the UK and internationally. She was awarded a BABCP Honorary Fellowship in 2022. The articles collated in this special collection demonstrate the depth and range of her expertise, as well as her talent for explaining complex ideas in understandable and clinically applicable ways. Over time, we also hope to add further papers from other researchers and clinicians whose contributions have been directly inspired by Hannah’s work. In a special symposium at the BABCP conference in 2024, we celebrated Hannah’s many contributions to the field, with reflections from friends, colleagues, and collaborators that have informed the present article. We presented the inaugural ‘tCBT Hannah Murray Award for the Best Practitioner Paper of the Year’ to Mitchell and Allen’s (2023) paper on adapting CBT for depression in later life for Irish Travellers. Further information on this award is available at: https://www.cambridge.org/core/journals/the-cognitive-behaviour-therapist/announcements/prizes-and-awards

This Editorial aims to document and celebrate Hannah’s work. In line with Hannah’s mission to support clinicians to develop their confidence and skills in treating PTSD, we aim to extract some of the key clinical tips and principles from each area of her work, and direct readers to the excellent resources she has developed with her many colleagues and collaborators. We write this on behalf of all those who had the honour of working with Hannah, whether in her role as therapist, trainer, supervisor, researcher, colleague, or friend.

### CT-PTSD treatment delivery and its misconceptions

Cognitive therapy for PTSD (CT-PTSD) is a type of trauma-focused cognitive behavioural therapy that is recommended as a first-line treatment for PTSD in UK (NICE, 2018) and international guidelines (International Society of Traumatic Stress Studies, 2019). Hannah was a true expert in this treatment, and contributed to innovative studies exploring the effectiveness of different treatment formats, such as intensive treatment delivered over 5 days (Murray *et al*., 2017), and internet-delivered treatment (Ehlers *et al*., 2023). She also helped develop guidance on how to deliver CT-PTSD when working remotely (Wild *et al*., 2020), highlighting small adaptations to consider when implementing each technique.

It is understandable that therapists who are new to (or feel less confident about) treating PTSD may feel apprehensive, perhaps being concerned that talking about the trauma may be retraumatising for the patient, or believing that a period of stabilisation is always necessary before trauma-focused work. Based on these beliefs, therapists may omit or delay critical CT-PTSD techniques such as imaginal reliving of the trauma memory. Murray and colleagues’ (2022a) paper explains why these two beliefs, and eight other commonly held ideas about CT-PTSD, are actually misconceptions, and what should be done instead. A core principle Hannah would highlight is to get going with work on the trauma memory as soon as possible, because the evidence shows it is one of the most powerful components of treatment. We have been told this paper is now considered essential reading on several CBT training courses.

One powerful method to help practitioners feel more confident trying a new technique is to show them what it looks like. Together with her colleagues at the Oxford Centre for Anxiety Disorders and Trauma, Hannah made multiple short video clips demonstrating key CT-PTSD techniques. These are freely available at www.oxcadatresources.com and at the time of writing have been viewed by over 47,000 users in 175 countries.

### Behavioural experiments, surveys, and site visits

Techniques in CT-PTSD where the patient and therapist try doing something together in the session can be some of the most clinically important components of therapy. These include behavioural experiments (e.g. to test out a patient’s feared concern, or explore the potential negative consequences of safety behaviours), surveys (to understand what others may be thinking in relation to the patient’s situation), and visits back to the site of the trauma. Despite their clinical value, data from clinicians working in NHS Talking Therapies attending a top-up training course in CT-PTSD showed that these are some of the least frequently used techniques (Warnock-Parkes *et al*., 2024).

Hannah would frequently emphasise the idea that ‘*CBT is a doing therapy, not just a talking therapy*’. She would encourage all practitioners to make their sessions more active and helped develop specific guidance on doing this in CT-PTSD. Murray and El-Leithy (2021) describe why behavioural experiments are so valuable in CT-PTSD, guiding readers on when they should be used and how to make them maximally effective. Guidance on using surveys of others’ views is given in Murray *et al*. (2022b), with a range of tips on writing good survey questions, administering them to the right people, and discussing the results. Conducting a visit back to the site of the trauma, either in-person or virtually (e.g. using Google Street View) is recommended for all patients in CT-PTSD where this is possible given the nature of the trauma. Murray *et al*. (2015) provide clear guidance on choosing the right time in treatment for this, practicalities to consider, and what to do while you are there. A related qualitative study describes patients’ experiences of doing this within their treatment (Murray *et al*., 2016).

### PTSD following Intensive Care Unit treatment

Studies have indicated that around a quarter of people admitted to an intensive care unit (ICU) show symptoms of PTSD between 1 and 6 months after discharge (Parker *et al*., 2015). ICU can be an overwhelming and frightening experience, particularly as patients will be acutely unwell and many on sedative medication. The Murray *et al*. (2020) paper outlines key information for practitioners working with ICU-related PTSD, such as how to work with hallucinations and delusions related to ICU medication, and applying CT-PTSD techniques to this type of trauma. Hannah also wrote about her own experiences in ICU with her characteristic warmth, wisdom and candour, for the BABCP magazine ‘CBT Today’ (Delirium Diaries: My Stay in ICU).

### Birth trauma and baby loss

It has been estimated that 4% of mothers go on to develop PTSD following traumatic experiences during childbirth (Yildiz *et al*., 2017), with around 30% reporting subthreshold symptoms (Creedy *et al*., 2000). In the Kerr *et al*. (2023) clinical guidance paper, Hannah and colleagues explain how the core elements of CT-PTSD can be applied in relation to birth trauma and baby loss. They highlight common cognitive themes linked to these types of trauma (e.g. an over-generalised sense of danger, an inflated sense of responsibility, and others), how these can be addressed in treatment and how to tailor treatment accordingly when treating somebody in the perinatal period.

### Moral injury

Moral injury refers to the significant distress that can develop following events where an individual’s moral code is transgressed. This may include betraying, harming or failing to help others, either directly or through witnessing or being subjected to such events. In their clinical guidance paper, Murray and Ehlers (2021) emphasise that although moral injury is commonly linked to veterans and occupational groups such as healthcare workers and journalists, it is not restricted to such groups and can be present across a range of people and trauma types. They outline how moral injury is conceptualised within the cognitive model of PTSD (Ehlers and Clark, 2000), and how to apply the core CT-PTSD treatment techniques.

### Survivor guilt

It is common that following a traumatic experience in which others died, those that survived report feeling guilty about doing so. Hannah demonstrated that 38.5% of those with PTSD in a UK clinic sample had experienced a trauma in which someone had died, that over 90% of these people reported experiences of survivor guilt, and that this was associated with more severe PTSD symptoms (Murray, 2018). Following qualitative work documenting the experience of survivor guilt (Pethania *et al*., 2018), and a proof-of-concept treatment study using imagery rescripting (Murray *et al*., 2021a), Hannah and colleagues proposed a cognitive approach to its conceptualisation and treatment (Murray *et al*., 2021b), highlighting that survivor guilt may be present in the absence of PTSD, and suggesting candidate maintenance processes that could inform treatment and future research.

### Complexity and complex PTSD

There are many factors that can add complexity to clinical presentations and the treatment of PTSD. These include co-morbid physical or mental health conditions, social and financial instability, clinical risk, issues of diversity, trauma history, therapeutic relationship challenges, as well as aspects of the trauma memory itself, trauma-related appraisals, and unhelpful coping behaviours. These complexity factors should not be confused with the recent formal definition of ‘complex PTSD’ (cPTSD) in the International Classification of Diseases 11th edition (World Health Organisation, 2019), which refers to the presence of ‘disturbances of self-organisation’ in addition to core symptoms of PTSD. Both PTSD and cPTSD may include complexity factors to a greater or lesser extent.

The book ‘*Working with Complexity in PTSD*’ (Murray and El-Leithy, 2022) systematically examines each of these complexity factors, and guides the practitioner on what to consider during treatment, and how to embrace and address these complexities when delivering CT-PTSD. It contains a wealth of clinical examples, extracts and tips, and represents one of Hannah’s finest achievements in her career. A key message throughout the book is that although complexity may require some flexibility regarding which techniques are used in which order, and how they are implemented, it is important to do this within the context of the core treatment model and principles of CT-PTSD. As Hannah herself put it:

‘Within the framework of a collaborative and supportive therapeutic relationship, and sticking closely to the individual case formulation derived from Ehlers and Clark’s (2000) cognitive model, we encourage therapists to be curious, creative and confident in addressing the memories and meanings that are central to the experience of PTSD’ *(Murray et al., 2022a, p. 12)*

## Conclusion

We hope that this Editorial will serve as a resource to summarise and guide practitioners towards the wealth of resources Hannah has been involved in creating, and stand as a celebration of a remarkable talent in our field. We remain truly sad to have lost Hannah but can take comfort from her incredible legacy. We are confident that this body of work will continue to inspire and guide current and future clinicians in their practice for many years to come.

## Data Availability

Data availability is not applicable to this article as no new data were created or analysed in this study.
